# A prospective feasibility study of a 1-mm bolus for postmastectomy radiotherapy

**DOI:** 10.1186/s12885-021-07851-3

**Published:** 2021-02-02

**Authors:** Terufumi Kawamoto, Naoto Shikama, Chie Kurokawa, Naoya Hara, Masaki Oshima, Keisuke Sasai

**Affiliations:** 1 Department of Radiation Oncology, Juntendo University, Graduate School of Medicine, 2-1-1 Hongo, Bunkyo-ku, Tokyo, 113-8421 Japan; 2 Department of Radiology, Juntendo University Hospital, Tokyo, Japan

**Keywords:** Bolus, Breast cancer, Dosimetry analysis, Postmastectomy radiotherapy

## Abstract

**Background:**

The optimal chest wall bolus regimen for postmastectomy radiotherapy (PMRT) remains unknown. We aimed to prospectively evaluate the use of a 1-mm-thick daily tissue-equivalent bolus in patients who received PMRT using thermoluminescent dosimeters (TLDs) and skin toxicity assessment.

**Methods:**

Patients with a 1-mm-thick daily bolus during PMRT were prospectively enrolled at The Juntendo University Hospital. The surface dose was measured in vivo under the 1-mm-thick bolus on the chest wall. We assessed the acute skin toxicity weekly during PMRT, and 1, 2, 4, and 12 weeks after the completion of PMRT.

**Results:**

A total of 19 patients aged 32–79 years old received PMRT from July 2019 to January 2020. All patients completed the protocol treatment without interruptions, and the median follow-up was 32 weeks. In vivo dosimetry analysis revealed surface doses between 77 and 113% of the prescribed dose, with a mean of 92% of the prescribed radiation dose, and a standard deviation of 7% being delivered. Grade 2 dermatitis was found in 10 patients (53%), and Grade 3 dermatitis was found in one patient (5%). All cases of Grade 2 and 3 dermatitis were improved 4 weeks after PMRT. There were no cases of Grade 4 dermatitis and no chest wall recurrences during the treatment or follow-up period.

**Conclusions:**

Results confirmed the feasibility of using a 1-mm-thick daily bolus for PMRT, exhibiting an appropriate dose buildup and acceptable skin toxicity without treatment interruptions.

**Trial registration:**

The University Hospital Medical Information Network Clinical Trials Registry, UMIN000035773. Registered 1 July 2019.

## Background

Postmastectomy radiotherapy (PMRT) has proven useful for increasing both locoregional control and overall survival among patients with high-risk breast cancer [1–4]. However, there are currently various options regarding the bolus regimen and bolus material for PMRT.

Tissue-equivalent materials are applied to the chest wall to provide dose buildup in the skin and tissue in order to adequately deliver the prescription dose to the level of skin and treat residual disease. Previous surveys have reported that 50–90% of institutions use such tissue-equivalent boluses [5–7]. The most common practice in a reported survey was a 5-mm-thick bolus on alternate days [7]. However, this method carries the risk of clinical error and the possibility that the bolus is not used on the set days. In addition, it requires two treatment plans that extend the duration, and induce further potential for error. By contrast, the use of daily boluses makes it easier to assess efficacy and toxicity since the daily dose is always the same; although this method increases the risk of severe skin toxicity [8]. As a basic experiment in our hospital, we measured the skin dose under the 1-, 2-, 3-, or 5-mm bolus on the chest wall using a phantom. We confirmed the previous finding that the mean surface dose of a 1-mm-thick daily bolus approximated that of a half-time 5-mm-thick bolus [9].

Thus, we hypothesized that a 1-mm-thick daily bolus would be a feasible regimen to reduce skin toxicity without the need for two treatment plans. Here we report the results of skin toxicity associated with PMRT using a 1-mm-thick daily bolus, as well as the skin dose using thermoluminescent dosimeters (TLDs; Toyo Medic Co., Ltd.; Tokyo, JP).

## Methods

### Patient selection

Patients (i) with pathologically confirmed breast cancer; (ii) requiring PMRT; (iii) aged 20 years and older; (iv) with an Eastern Cooperative Oncology Group performance status (ECOG PS) of 0–2; and (v) with no history of prior overlapping radiation were eligible for the study. Patients (i) with placement of a tissue expander, implant, or autologous tissue reconstruction; (ii) requiring boost irradiation for positive surgical margin; (iii) with severe comorbidity; and (iv) active double cancer were excluded from the study.

### Study design

This single-center feasibility study evaluated the use of a 1-mm-thick daily tissue-equivalent bolus in patients who received PMRT using TLDs and skin toxicity assessment. As a pilot study, we aimed to recruit 20 participants for assessment of the surface dose and skin toxicity. Written informed consent was obtained from all patients. This prospective study was approved by the Institutional Review Board at The Juntendo University Hospital (approval number: 19–087), and was registered at the University Hospital Medical Information Network Clinical Trials Registry (UMIN000035773).

### Treatment

Treatment plans were generated from a Toshiba Aquilion 16 LB computed tomography (CT) scanner (Toshiba Medical Systems Inc.; Otawara, JP), with 3-mm slices from the upper neck to the mid-abdomen. Three-dimensional treatment planning was performed using the Eclipse treatment planning system (Varian Medical Systems Inc.; Palo Alto, USA) TPS v. 13.6. All patients were treated with tangential 6-MV photon beams to the chest wall using the field-in-field technique, and a combination of anterior and posterior oblique 6- or 10-MV photon beams for the supraclavicular fossa. Patients with clinically positive internal mammary nodes were treated with wide tangential 6-MV photon beams to both the chest wall and internal mammary node area. Dose calculation was performed with an anisotropic analytical algorithm. Chest wall separation was measured between the sternal beam entry and the midaxillary line.

**Fig. 1:**
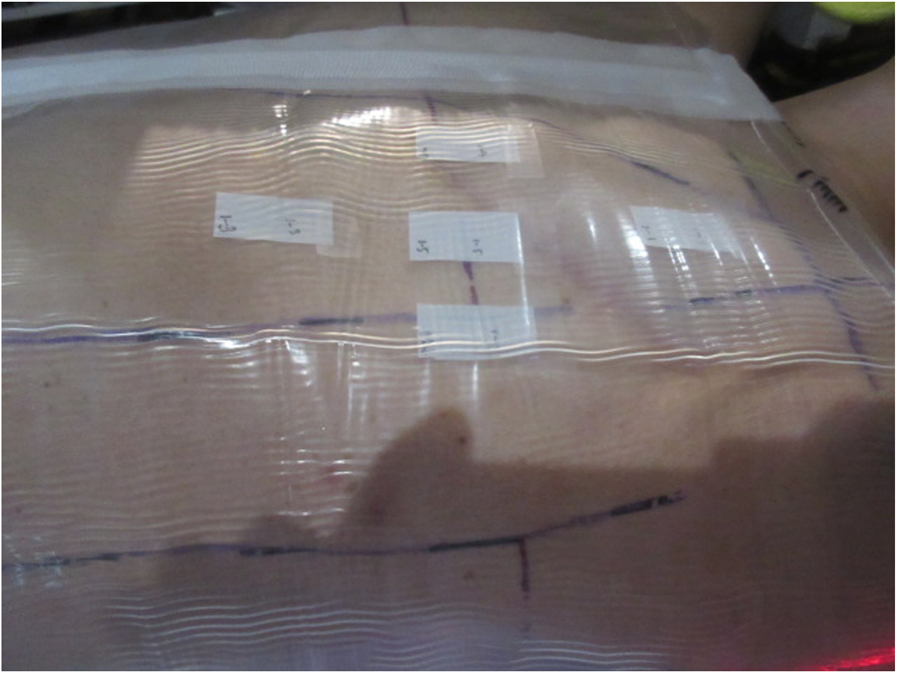
In vivo chest wall standard positions

A 1-mm-thick tissue-equivalent Clearfit bolus (Fujidenolo Inc.; Aichi, JP) was placed over the chest wall, daily, for the entire course of PMRT (Fig. 1). The Clearfit bolus for the linac was colorless and soft, with a transparent finish. The daily radiotherapy fraction was 2.0 Gy according to the International Commission on Radiation Units and Measurements reference point, and was administered 5 days per week for a total dose of 50 Gy. Radiation plans were assessed by evaluating both isodose lines and the dose-volume histogram. A maximum hotspot of 115% was allowed.

### In vivo dosimetry

In all patients, the surface dose to the skin under the bolus was measured within 5 days of beginning PMRT. Based on previous study, TLDs were placed on the chest wall by one radiation oncologist, and measurements were taken at five sites as follows: Central, medial, lateral, superior, and inferior (Fig. 1) [10]. The first TLD was placed at the center of the field light. With this point as the center, each TLD was placed 3 to 5 cm to the medial, lateral, superior, and inferior. After irradiation, TLD was read out using a TLDR-1 (Toyo Medic CO., LTD., Tokyo, JP), and calculated using the DoseLab (Mobius medical system LP; Houston, USA). A 5 × 5 mm area of each TLD was used to determine the mean dose and standard error.

### Skin toxicity

Skin toxicity was evaluated weekly during PMRT, and 1, 2, 4, and 12 weeks after completion of PMRT by two radiation oncologists. Radiation dermatitis was scored using the National Cancer Institute Common Terminology Criteria for Adverse Events (NCI-CTCAE) version 5.0 [11]. Standard skin care included nonfragrant moisturizing cream for skin dryness, and steroid cream in cases of excessive redness or inflammation.

## Results

### Patient characteristics

Of the 20 enrolled patients, one refused to participate; thus, 19 patients were enrolled from July 2019 to January 2020. All patients satisfied the eligibility criteria, and the patient characteristics are presented in Table 1. All patients completed the protocol treatment without interruptions, and the median treatment period was 35 (range, 32–40) days. The median follow-up from the start of PMRT was 32 (range, 19–54) weeks.

**Table 1:** Patient and tumor characteristics

Patient and tumor characteristics	No. (%)
Median age, years (range)	59 (32–79)
Median height, cm (range)	155 (150–179)
Median weight, kg (range)	55 (43–74)
Median BMI (range)	21.8 (17.6–28.3)
History of smoking	
None	14 (74)
Former smoker	5 (26)
Diabetes	
Yes	1 (5)
No	18 (95)
High blood pressure	
Yes	4 (21)
No	15 (79)
Cardiovascular disease	
Yes	0 (0)
No	19 (100)
Collagen disease	
Yes	0 (0)
No	19 (100)
ECOG PS	
0	18 (95)
1	1 (5)
Laterality	
Left	9 (47)
Right	10 (53)
Clinical T stage	
1b	1 (5)
1c	3 (16)
2	11 (58)
3	3 (16)
4b	1 (5)
Clinical N stage	
0	2 (11)
1	9 (47)
2a	3 (15)
2b	1 (5)
3a	2 (11)
3b	2 (11)
Clinical stage group	
IA	1 (5)
IIA	4 (21)
IIB	6 (32)
IIIA	4 (21)
IIIB	1 (5)
IIIC	3 (16)
Pathologic T stage	
0	4 (21)
1a	2 (11)
1c	4 (21)
2	6 (31)
3	2 (11)
4b	1 (5)
Pathologic N stage	
0	3 (16)
1mi	2 (11)
1a	8 (42)
2a	5 (26)
3a	1 (5)
Pathologic stage group	
pCR	2 (11)
IA	1 (5)
IB	2 (11)
IIA	2 (11)
IIB	4 (21)
IIIA	7 (36)
IIIC	1 (5)
Receptor status	
ER positive	14 (74)
ER negative	5 (26)
PR positive	11 (58)
PR negative	8 (42)
HER2 positive	12 (64)
HER2 negative	7 (36)
Surgical margin	
Close	4 (21)
Negative	15 (79)
Chemotherapy delivery	
Neoadjuvant	10 (53)
Adjuvant	8 (42)
No	1 (5)
Hormone therapy	
Yes	14 (74)
No	5 (26)
Concurrent hormone therapy (yes)	10 (53)
Anti-HER2 therapy (trastuzumab + pertuzumab)	
Yes	8 (42)
No	11 (58)
Concurrent Anti-HER2 therapy (yes)	7 (36)
Median chest wall separation, cm (range)	19.0 (16.4–22.3)

*Abbreviations*: *BMI* Body mass index, *ECOG PS* Eastern Cooperative Oncology Group performance status, *ER* Estrogen receptor, *PR* Progesterone receptor. Values are number (percentage) or median (range)

### In vivo dosimetry

The TLDs measurements are shown in Table 2. In vivo dosimetry analysis revealed surface doses between 77 and 113% of the prescribed dose, with a mean of 92% of the prescribed radiation dose, and a standard deviation of 7% being delivered. The mean percentages of the prescribed radiation dose (standard deviation) for the central, medial, lateral, superior, and inferior TLDs were 93% (5%), 86% (5%), 98% (7%), 89% (5%), and 94% (5%), respectively.

**Table 2:** TLD measurements on the skin underneath the 1-mm chest wall bolus

	Mean % of prescribed dose (range; SD)
Central	93% (80–104%; 5%)
Medial	86% (77–96%; 5%)
Lateral	98% (88–113%; 7%)
Superior	89% (80–100%; 5%)
Inferior	94% (86–105%; 5%)
Total	92% (77–113%; 7%)

*Abbreviations*: *TLD* Thermoluminescent dosimeter, *SD* Standard deviation

### Skin toxicity

**Fig. 2:**
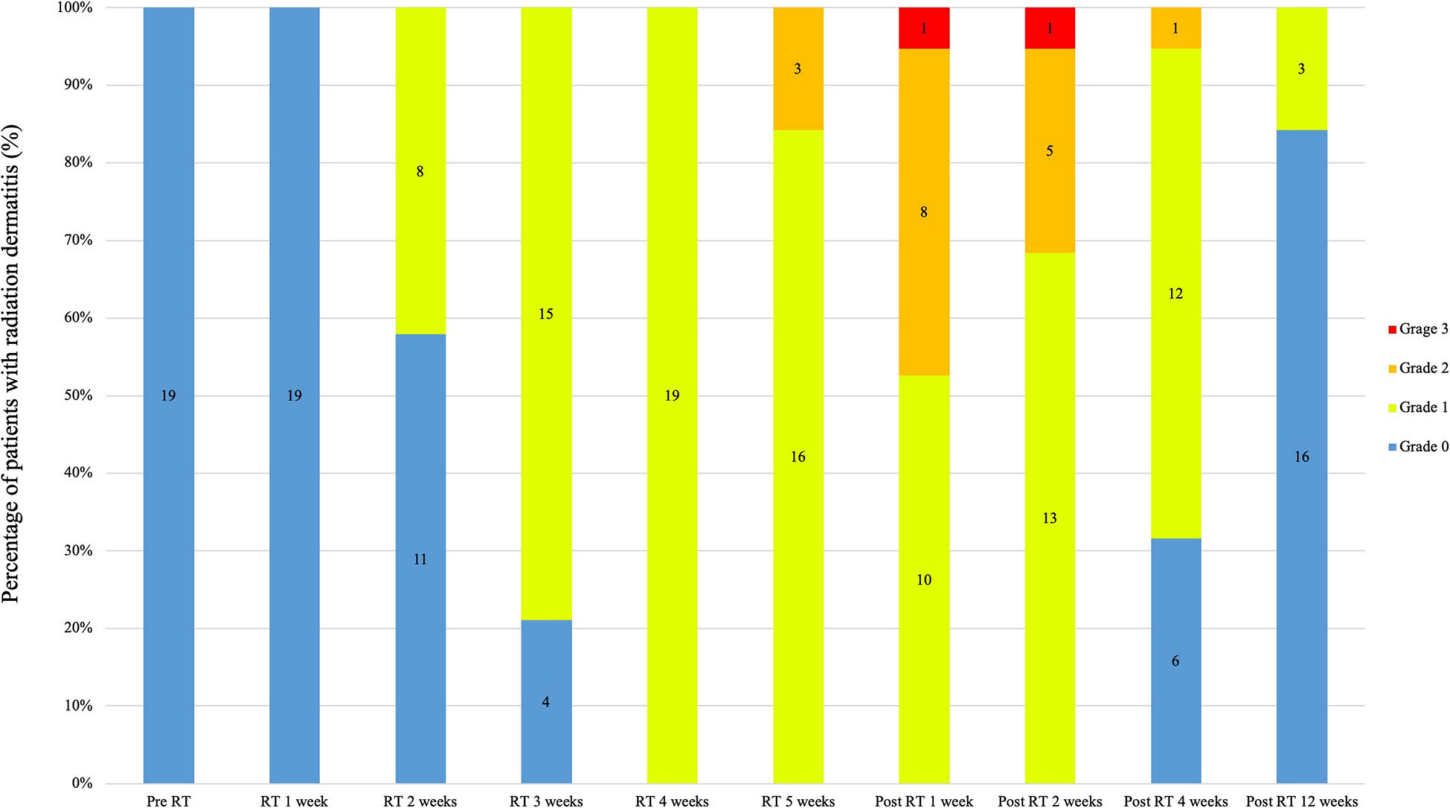
Skin toxicity data. Abbreviation: RT: Radiotherapy

All patients experienced radiation dermatitis during the treatment and follow-up period (Table 3, Fig. 2). Grade 2 dermatitis was found in 10 patients (53%) during, or within 2 weeks of PMRT. Grade 3 dermatitis (moist desquamation of the chest wall) was found in one patient (5%) 1 week after PMRT. All cases of Grade 2 and 3 dermatitis were improved 4 weeks after PMRT. No patient developed Grade 4 dermatitis.

**Table 3:** Maximum radiation dermatitis measured during the treatment and follow-up period

Skin toxicity score	No. (%)
0: No skin changes	0 (0)
1: Faint erythema or desquamation	8 (42)
2: Moderate to brisk erythema; patchy moist desquamation, mostly confined to skin folds and creases; and moderate edema	10 (53)
3: Moist desquamation in areas other than skin folds and creases, and bleeding induced by minor trauma or abrasion	1 (5)
4: Life-threatening consequences: skin necrosis or ulceration of full thickness dermis, spontaneous bleeding from the involved site, skin graft indicated	0 (0)

### Clinical outcomes

No patient exhibited chest wall recurrence during the treatment and follow-up period. One patient was found to have metastatic diseases to the bone and liver 4 weeks after PMRT.

## Discussion

Dosimetric studies using a thinner daily bolus are limited in the existing literature. In one study, Healy et al. reported the surface dose measurements of 16 patients using a daily 2-mm brass-mesh bolus for PMRT recorded by TLDs. They reported mean surface doses between 81 and 122% of the prescribed dose, with a mean of 99% of the prescribed radiation dose, and a standard deviation of 10% being delivered [12]. Another study reported that surface doses among no bolus, a 1-mm-thick brass-mesh bolus, a 3-mm-thick Superflab bolus, and a half-time 10-mm-thick Vaseline bolus using a phantom recorded by TLDs were 68, 91.6, 97.7, and 100.7%, respectively [13]. Taken together, these results suggest that a brass-mesh bolus might be beneficial for PMRT; however, its use is limited in our country because of the off-label use. The dosimetric outcomes of the present study are similar to those of previously published data, in that the mean surface dose was 92% (range, 77–113%) of the prescription dose. Therefore, it is conceivable that a mean surface dose of approximately 90% of the prescribed dose (45 Gy in 25 fractions) was appropriate dose build up.

Our study demonstrated that the 1-mm-thick daily bolus is a safe regimen for PMRT with skin toxicity without treatment interruptions. In our study, only one (5%) patient was found to have Grade 3 skin toxicity, which is adequate compared to the published data of 12.2% with a daily 2-mm bolus, and 2.1% with no bolus [10, 14]. Indeed, Tieu et al. reported that PMRT was ceased early because of unacceptable skin toxicity in 17/143 (12%) of the whole chest wall daily 10-mm bolus patients, 2/88 (2%) of the parascar daily 10-mm bolus patients, and 1/23 (4%) of the no bolus patients. They concluded that the use of daily boluses may impact on early cessation of PMRT caused by skin toxicity, which may subsequently influence chest wall recurrence [8]. With regards to treatment completion with the daily 1-mm bolus, it is possible that it will influence chest wall control. However, our finding that there were no cases of chest wall recurrence should be viewed with caution given the short follow-up period.

Maximum skin toxicity can occur 1 to 2 weeks after completion of PMRT [15]. However, previous prospective studies using a thinner daily bolus only reported skin toxicity during PMRT [10, 12]. We improved the research accuracy by evaluating skin toxicity 1, 2, 4, and 12 weeks after completion of PMRT in all patients.

In our study, 10 (53%) patients received PMRT concurrent with hormone therapy, and seven (36%) patients received PMRT concurrent with a combination of pertuzumab and trastuzumab. A recent meta-analysis concluded that PMRT concurrent with hormone therapy showed no significant difference in the incidence of radiation-induced acute skin toxicity compared to that of the sequential group [16]. However, the safety of PMRT concurrent with combination of pertuzumab and trastuzumab remains unclear [17]. At least, in our study, PMRT concurrent with a combination of pertuzumab and trastuzumab was well tolerated.

This study has several limitations. First, the sample size was small and the follow-up time was short. The follow-up period was used to determine acute skin toxicity, but it was not adequate to observe late toxicities. Second, although the use of the NCI-CTCAE skin toxicity scoring system attempts to standardize the quantification of skin toxicity, a small amount of subjectivity is required to assign scores; thus, assigned scores may vary between radiation oncologists. However, this difference is likely to be small given that skin toxicity was evaluated by two radiation oncologists in our study. Third, our study excluded patients with a positive surgical margin and those who underwent reconstruction. There are wide differences in the practice patterns regarding the use of a PMRT boost for margin status [7]. In our hospital, the decision to administer a boost for a total dose of 10 Gy with a photon or electron beam was influenced by several factors, including positive surgical margins. We therefore examined radiation dermatitis using the same dose and conditions in this study. Moreover, we excluded patients who underwent reconstruction because reconstruction tended to be a significant predictor of moist desquamation [18]. Fourth, the efficacy of the 1-mm-thick daily bolus for PMRT remains unclear because of the short follow-up period. Our findings of no chest wall recurrence should be viewed with caution. We will assess chest wall recurrence rates in our future study.

## Conclusions

Our results confirmed the feasibility of using a 1-mm-thick daily bolus for PMRT, with appropriate dose buildup and acceptable skin toxicity without treatment interruptions.

This regimen could omit the clinical error and potential failure associated with using two treatment plans and bolus on alternate days while also making it easy to assess efficacy and toxicity since the daily dose is the same.

## Data Availability

The datasets used and/or analyzed during the current study are available from the corresponding author on reasonable request.

## Abbreviations

CT: Computed tomography
ECOG PS: Eastern Cooperative Oncology Group performance status
NCI-CTCAE: National Cancer Institute Common Terminology Criteria for Adverse Events
PMRT: Postmastectomy radiotherapy
TLD: Thermoluminescent dosimeter
